# Osimertinib for EGFR‐Mutant NSCLC Patients With Acquired T790M and EGFR Amplification After First‐Generation EGFR‐TKI Resistance

**DOI:** 10.1111/cas.16437

**Published:** 2024-12-31

**Authors:** Yidan Zhang, Yingqi Xu, Jianlin Xu, Hua Zhong, Jinjing Xia, Runbo Zhong

**Affiliations:** ^1^ Department of Respiratory and Critical Care Medicine, Shanghai Chest Hospital Shanghai Jiao Tong University School of Medicine Shanghai China

**Keywords:** EGFR, EGFR amplification, EGFR‐TKI, NSCLC, T790M

## Abstract

Third‐generation epidermal growth factor receptor tyrosine kinase inhibitor (EGFR‐TKI) is the standard therapy for patients harboring T790M after first‐generation EGFR‐TKI resistance. However, the impact of acquired EGFR amplification on the efficacy of third‐generation EGFR‐TKI against T790M remains uncertain. We aimed to investigate whether the presence of acquired EGFR amplification after first‐generation EGFR‐TKI resistance influences the efficacy of third‐generation EGFR‐TKI in patients with advanced non‐small‐cell lung cancer (NSCLC). We reviewed data from 275 advanced NSCLC patients harboring T790M after first‐generation EGFR‐TKI resistance. Patients were categorized into two groups based on the presence or absence of acquired EGFR amplification identified through next‐generation sequencing (NGS) after first‐line EGFR‐TKI treatment. We evaluated the efficacy of osimertinib used as a second‐line treatment. Among these patients, 59 exhibited acquired EGFR amplification, while 216 did not. The median progression‐free survival (PFS) was 12.20 months in the EGFR amplification group and 12.03 months in the non‐amplification group (*p* = 0.011), with median overall survival (OS) of 33.90 months and 23.30 months, respectively (*p* = 0.164). Multivariate analysis of PFS revealed that acquired EGFR amplification and EGFR 19del were independent prognostic factors for patients with T790M undergoing osimertinib. Additionally, subgroup analysis indicated a prolonged PFS in patients with EGFR 19del compared to those with EGFR 21L858R (*p* = 0.034) in the EGFR amplification group. Following first‐generation EGFR‐TKI resistance, advanced EGFR‐mutant NSCLC patients harboring both acquired T790M and EGFR amplification are likely to experience enhanced PFS with osimertinib. This phenomenon is particularly noteworthy among individuals with EGFR 19del.

## Graphical abstract

Acquired EGFR amplification may coincide with T790M after first‐generation EGFR‐TKI resistance, accounting for 21.5% of cases in our study. Patients with both acquired T790M and EGFR amplification after first‐generation EGFR‐TKI may derive enhanced benefits from 3rd EGFR‐TKI. Patients with both acquired T790M and EGFR amplification, particularly those with EGFR 19del, exhibited a longer PFS.

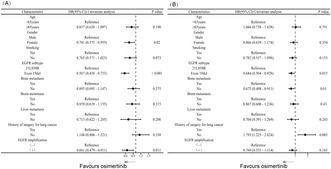

## 1 Introduction

Non‐small cell lung cancer (NSCLC) accounts for approximately 80%–85% of all lung cancer patients, with a majority diagnosed at advanced stages. Furthermore, lung adenocarcinoma (LUAD) is predominant among NSCLC subtypes. Previous studies have shown a high prevalence of epidermal growth factor receptor (EGFR) mutations, impacting approximately 11% of Caucasian and 50% of Asian LUAD patients. All three generations of EGFR‐TKIs serve as standard first‐line treatments for NSCLC patients with EGFR‐sensitive mutations, primarily comprising exon 19 deletions (19del) and the L858R point mutation in exon 21 (21L858R) [1, 2, 3, 4]. However, most patients develop resistance after 10–14 months of treatment with first‐ or second‐generation EGFR‐TKI. Resistance mechanisms primarily include EGFR‐dependent mechanisms, EGFR‐independent mechanisms, and histological transformation [5]. The T790M mutation is the most prevalent mechanism of acquired resistance to first‐ or second‐generation EGFR‐TKI, representing a resistance mutation dependent on the EGFR pathway, with an incidence ranging from approximately 50% to 60% [6]. Multiple third‐generation EGFR‐TKIs have been developed in response to the acquired T790M mutation, yielding median PFS durations ranging from 9.6 to 12.4 months and median OS spanning from 22.1 to 30.2 months [6, 7, 8, 9, 10, 11, 12].

EGFR amplification is typically defined as an EGFR gene/chromosome per cell ratio ≥ 2, or ≥ 15 copies of the genes per cell in ≥ 10% of analyzed cells [13]. EGFR amplification often occurs concurrently with EGFR mutations [14, 15, 16]. Moreover, the coexistence of EGFR amplification and EGFR mutations before targeted agent therapy typically predicts a favorable response to first‐generation EGFR‐TKI treatment [14]. Interestingly, emerging evidence suggests that the development of EGFR amplification after EGFR‐TKI treatment may serve as one of the acquired resistance mechanisms in EGFR‐mutant patients receiving EGFR‐TKI treatment [17, 18, 19]. After initiation of first‐generation EGFR‐TKI treatment, acquired EGFR amplification may coincide with the presence of the T790M mutation [20, 21]. Nonetheless, the impact of acquired EGFR amplification on the efficacy of third‐generation EGFR‐TKI as a second‐line treatment against acquired T790M mutation remains uncertain.

Therefore, this study aimed to investigate the potential impact of acquired EGFR amplification on the efficacy of third‐generation EGFR‐TKI monotherapy in patients with advanced NSCLC harboring EGFR‐sensitive mutations who developed the T790M mutation following resistance to first‐line EGFR‐TKI therapy.

## 2 Materials and Methods

### 2.1 Patients

**FIGURE 1:**
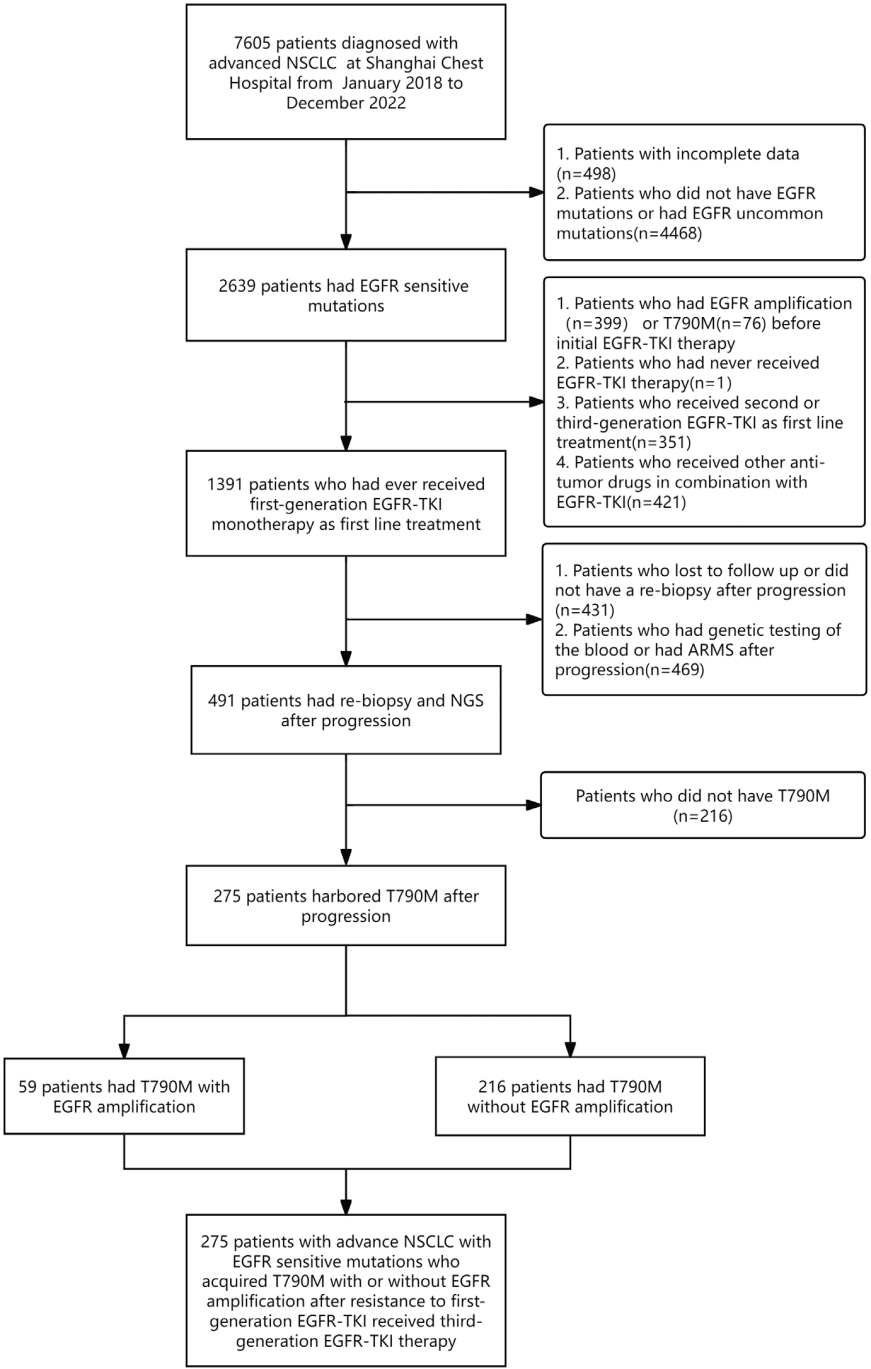
Flow diagram showing the screening of patients with advanced NSCLC with EGFR sensitive mutations who had T790M mutation with or without EGFR amplification after first‐generation EGFR‐TKI.

In this retrospective study, the medical records of 7605 patients diagnosed with advanced NSCLC at Shanghai Chest Hospital between January 2018 and December 2022 were reviewed. Inclusion criteria comprised patients meeting the following conditions: (I) harbored EGFR sensitive mutations (19del and 21 L858R) before initial EGFR‐TKI treatment, (II) experienced disease progression after first‐generation EGFR‐TKI treatment, (III) underwent re‐biopsy and NGS after disease progression, and (IV) acquired T790M mutation after first‐generation EGFR‐TKI treatment. The exclusion criteria encompassed patients with EGFR amplification identified before initial treatment, those not receiving first‐generation EGFR‐TKI as first‐line therapy, patients not undergoing re‐biopsy after acquiring resistance to first‐generation EGFR‐TKI, those with undetected T790M mutation upon re‐biopsy after disease progression despite first‐line treatment, patients whose re‐biopsy genetic testing was not performed using NGS, and individuals lost to follow‐up during the treatment of first‐generation EGFR‐TKI therapy or with incomplete data (see Figure 1). Ultimately, the study included 275 advanced EGFR‐mutant NSCLC patients with T790M mutation, with or without EGFR amplification detected by NGS after resistance to first‐generation EGFR‐TKI treatment. Subsequently, all these patients received monotherapy with the third‐generation EGFR‐TKI osimertinib.

### 2.2 Genetic Testing

NGS data may help to identify single nucleotide variants, insertions/deletions, copy number variations, and translocations at a single time point. Validated protocols are available for whole genome sequencing data and hybridization‐based targeted enrichment sequencing data sets [22, 23]. Following disease progression despite first‐generation EGFR‐TKI therapy, all the included patients of the present study underwent re‐biopsy and NGS for genetic analysis.

For pre‐processing of NGS, TrimmomaticPE was used to remove adapter sequences, and low‐quality sequences or sequences shorter than 50 bp were filtered out (parameter: ILLUMINACLIP: adapter. fa: 2: 30: 10: 1: true, TRAILING: 20, SLIDINGWINDOW: 30: 25, MINLEN:50). The Illumina TruSeq Amplicon Cancer Panel kit (Burning Rock Company, China) was utilized for library preparation and sequencing was performed on the MiSeq instrument. With regard to hybridization capture enrichment, the 68 genes with high mutation rates in lung cancer were added (see Table S1).

In this study, the cutoff value for EGFR amplification was defined as an EGFR copy number (CN) of ≥ 4, corresponding to an EGFR gene/chromosome per cell ratio ≥ 2.

### 2.3 Clinical Assessments and Follow‐Up

All patients were restaged according to the 8th edition of the tumor, node, metastasis (TNM) classification system before administering the third‐generation EGFR‐TKI. Over the therapeutic course, treatment efficacy was assessed by chest computed tomography (CT) scan every 2–3 months, with additional abdominal ultrasound, cranial magnetic resonance imaging (MRI), and bone emission computed tomography (ECT) if necessary. Treatment efficacy was monitored until disease progression, treatment discontinuation, or the last follow‐up, whichever occurred first.

The treatment response was evaluated based on Response Evaluation Criteria in Solid Tumors (RECIST, version 1.1). Responses to therapy were classified as complete response (CR), partial response (PR), stable disease (SD), or progressive disease (PD). In addition, the main assessment indicators included objective response rate (ORR), disease control rate (DCR), PFS, and OS. The last follow‐up visit for this study was on January 31, 2024.

### 2.4 Statistical Analysis

Statistical analyses were performed using SPSS 27.0 statistical software (IBM, Armonk, NY). The ORR was defined as the sum of the CR and PR proportions. DCR was the sum of the CR, PR, and SD proportions. PFS was calculated from the initiation of third‐generation EGFR‐TKI therapy to the first occurrence of disease progression, regimen change, or last follow‐up visit. OS was calculated from the initiation of third‐generation EGFR‐TKI therapy to death or the last follow‐up visit, depending on which one occurred first. The age was treated as a continuous variable and expressed as the median, while other variables were categorical and presented as counts and percentages. Continuous variables were analyzed using the *t*‐test, while categorical variables were evaluated using Pearson's chi‐squared test or Fisher's exact test to assess the differences in baseline characteristics between the EGFR amplification group and the control group. The Kaplan–Meier method was used to estimate the median PFS, 6‐month PFS rate, 12‐month PFS rate, median OS, 12‐month OS rate, 24‐month OS rate, etc. The log‐rank test was employed to compare the two groups and a subgroup analysis was performed on the EGFR amplification group. Multivariate Cox regression analysis was conducted to identify significant factors associated with PFS and OS in patients with T790M mutation treated with third‐generation EGFR‐TKI. *p* < 0.05 (two‐sided) was considered statistically significant.

## 3 Results

### 3.1 Patient Characteristics

A total of 7605 patients diagnosed with advanced NSCLC were screened, identifying 275 patients with the T790M mutation through NGS detection. These patients underwent tissue rebiopsy after disease progression despite first‐generation EGFR‐TKI treatment. All patients included in the study were histologically confirmed to have LUAD upon rebiopsy pathological examination. The final analysis included two groups, namely the EGFR amplification group (*n* = 59) and the EGFR non‐amplification group (*n* = 216). Among all the included cases, 21.5% (59/275) of patients exhibited acquired EGFR amplification.

The characteristics of the two groups are summarized in Table 1. The median age of the 275 patients was 60 years (range: 32–82). The patients were predominantly female (59.6%), with no history of smoking (77.5%), and harbored the EGFR 19del mutation (63.3%). Following the failure of first‐generation EGFR‐TKI therapy, the sites of rebiopsy mainly included the lung, lymph nodes, and pleural effusion. Genetic biopsies conducted after first‐generation EGFR‐TKI resistance revealed the persistence of the original EGFR‐sensitive mutation and the acquisition of the T790M mutation in all patients. Among the 59 patients with acquired EGFR amplification, 36 (61.0%) were found to have the EGFR 19del mutation, while 23 (39.0%) had the EGFR 21L858R mutation. Among the 216 patients without acquired EGFR amplification, 138 (63.9%) had the EGFR 19del mutation, and 78 (36.1%) had the EGFR 21L858R mutation. Additionally, before third‐generation EGFR‐TKI treatment, 35.6% of patients in the EGFR amplification group had bone metastasis, 20.3% had brain metastasis, and 6.8% had liver metastasis. In comparison, in the non‐amplification group, the respective proportions of patients with bone metastasis, brain metastasis, and liver metastasis were 45.4%, 22.7%, and 6.5%. Apart from the significantly higher age in EGFR amplification group, the baseline characteristics were not biased between EGFR amplification group and non‐amplification group.

**TABLE 1:** Demographic and clinical characteristics of the patients before receiving third‐generation EGFR‐TKI.

Characteristics	T790M with EGFR amplification (*N* = 59) (%)	T790M without EGFR amplification (*N* = 216) (%)	*p*
Age (years)			
Median	66	59	< 0 0.001
Range	39–81	32–82	
Gender			
Male	22 (37.3)	89 (41.2)	0.587
Female	37 (62.7)	127 (58.8)	
Smoking			
Yes	14 (23.7)	48 (22.2)	0.806
No	45 (76.3)	168 (77.8)	
Bone metastases			
Yes	21 (35.6)	98 (45.4)	0.179
No	38 (64.4)	118 (54.6)	
Brain metastases			
Yes	12 (20.3)	49 (22.7)	0.701
No	47 (79.7)	167 (77.3)	
Liver metastases			
Yes	4 (6.8)	14 (6.5)	1.000
No	55 (93.2)	202 (93.5)	
History of surgery for lung cancer			
Yes	18 (30.5)	55 (25.5)	0.437
No	41 (69.5)	161 (74.5)	
EGFR subtype			
Exon 19del	36 (61.0)	138 (63.9)	0.685
21L858R	23 (39.0)	78 (36.1)	
Re‐biopsy site			
Lung	32 (54.24)	109 (50.46)	0.223
Lymph node	16 (27.12)	44 (20.37)	
Pleural effusion	11 (18.64)	63 (29.17)	

Abbreviations: EGFR, epidermal growth factor receptor tyrosine; EGFR‐TKI, epidermal growth factor receptor tyrosine kinase inhibitor.

### 3.2 Efficacy and Survival Analysis

After a median follow‐up of 33.43 months (95% confidence interval [CI]: 31.09–35.78), efficacy was measured in all patients receiving third‐generation EGFR‐TKI monotherapy. Among the EGFR amplification group (*n* = 59), 34 patients were evaluated as PR, 18 as SD, and 7 as PD. In the non‐amplification group (*n* = 216), 1 patient achieved CR, 104 were assessed as PR, 93 as SD, and 18 as PD. The ORR for the two groups was 57.6% and 48.6%, respectively (*p* = 0.220), and the DCR was 88.1% and 91.6%, respectively (*p* = 0.409).

**FIGURE 2:**
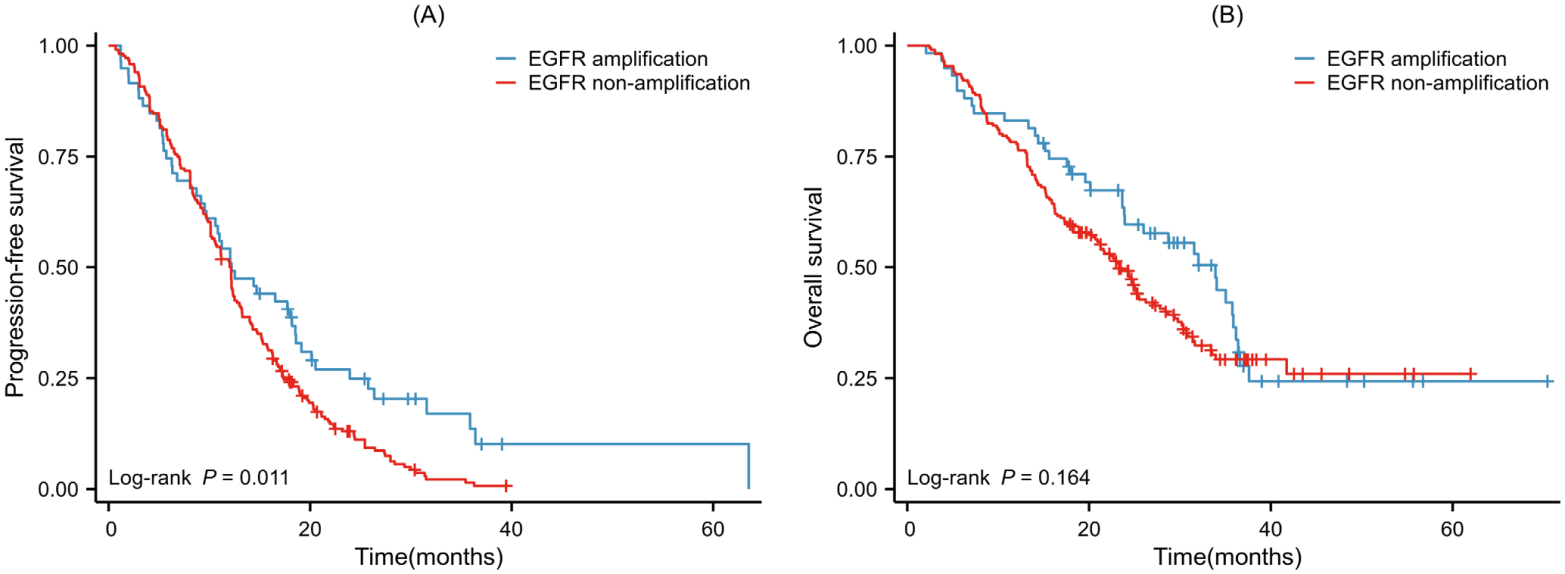
Kaplan–Meier estimates of the two groups. (A) Progression‐free survival and (B) Overall survival.

At the last follow‐up visit, 252 of 275 patients (91.3%) discontinued third‐generation EGFR‐TKI monotherapy due to disease progression. Notably, 49 (83.1%) were in the EGFR amplification group and 203 (94.0%) were in the non‐amplification group. The median PFS was 12.20 months (95% CI: 8.22–16.18) in the EGFR amplification group and 12.03 months (95% CI: 10.96–13.10) in the non‐amplification group (*p* = 0.011) (see Figure 2A). The 6‐month PFS rates in the EGFR amplification and non‐amplification groups were 72.9% and 78.2%, respectively, while the 12‐month PFS rates were 52.5% and 50.9%.

Among the 275 patients, 170 (61.8%) had died, with 36 deaths (83.1%) in the EGFR amplification group and 134 deaths (62.0%) in the non‐amplification group. The median overall survival (OS) was 33.90 months (95% CI: 26.93–40.87) for the EGFR amplification group and 23.30 months (95% CI: 20.84–25.76) for the non‐amplification group (*p* = 0.164) (see Figure 2B). The 1‐year OS rates for the amplification and non‐amplification groups receiving third‐generation EGFR‐TKI treatment were 79.7% and 77.8%, respectively, with 2‐year OS rates of 59.7% and 48.5%.

**FIGURE 3:**
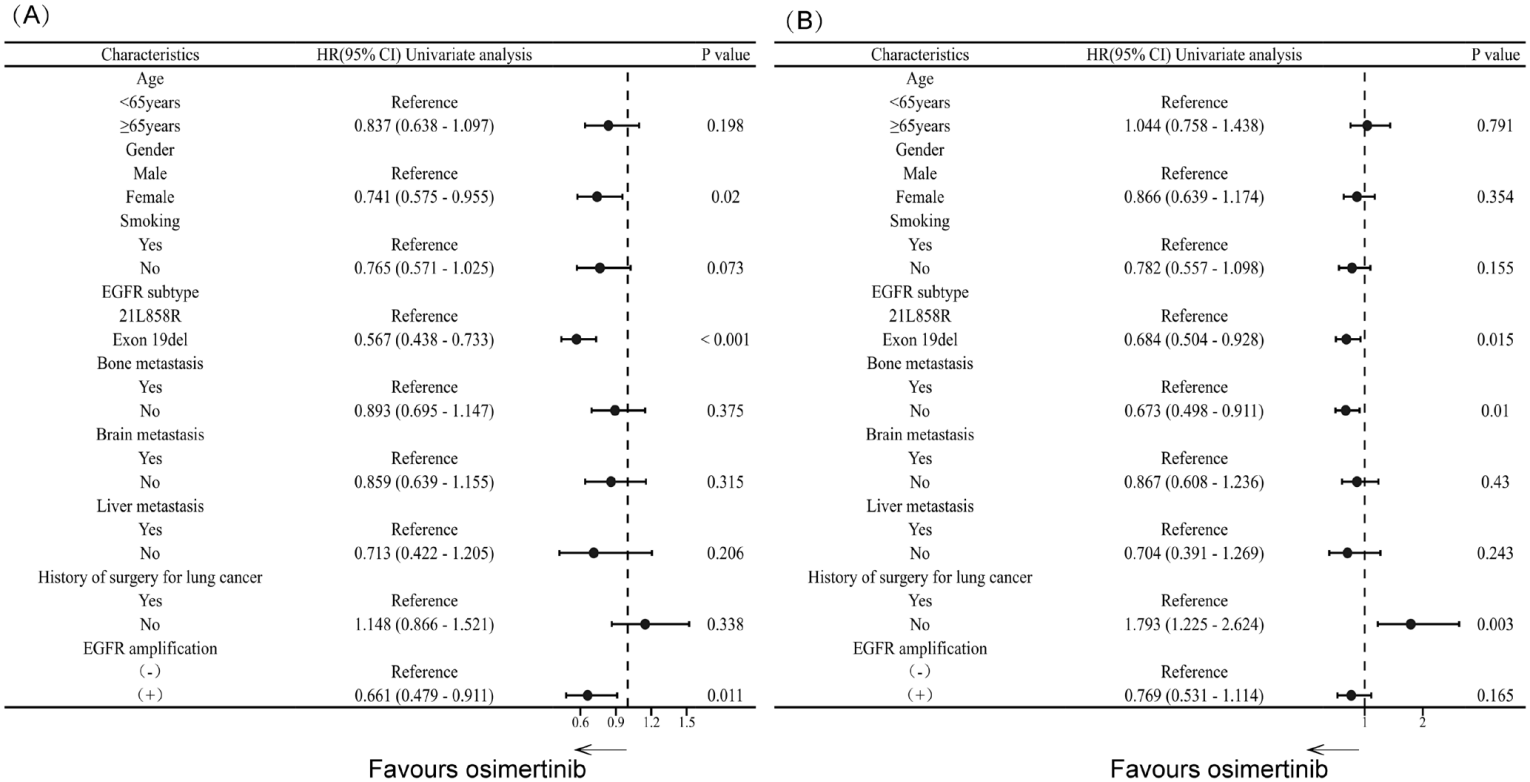
Cox regression hazard model related to (A) PFS and (B) OS of third‐generation EGFR‐TKI therapy. PFS, progression‐free survival; OS, overall survival.

Furthermore, univariate and multivariate analyses were conducted to identify factors influencing PFS and OS in all patients receiving third‐generation EGFR‐TKI treatment for T790M mutation. Univariate analysis revealed that acquired EGFR amplification [HR = 0.661, 95% CI (0.479–0.911), *p* = 0.011], female gender [HR = 0.741, 95% CI (0.575–0.955), *p* = 0.020], and EGFR 19del mutation [HR = 0.567, 95% CI (0.438–0.733), *p* < 0.001] were significantly associated with longer PFS (see Figure 3A). In addition, EGFR 19del mutation [HR = 0.684, 95% CI (0.504–0.928), *p* = 0.015], a history of lung cancer surgery [HR = 0.558, 95% CI (0.381–0.816), *p* = 0.003] and no bone metastasis [HR = 0.673, 95% CI (0.498–0.911), *p* = 0.010] were significantly associated with longer OS (see Figure 3B). Multivariate analysis revealed that acquired EGFR amplification [HR = 0.676, 95% CI (0.489–0.935), *p* = 0.018] and EGFR 19del mutation [HR = 0.561, 95% CI (0.433–0.726), *p* < 0.001] were independent prognostic factors for PFS, whereas EGFR 19del mutation [HR = 0.667, 95% CI (0.490–0.907), *p* = 0.010], a history of lung cancer surgery [HR = 0.611, 95% CI (0.415–0.900), *p* = 0.006] and no bone metastasis [HR = 0.703, 95% CI (0.516–0.958), *p* = 0.026] were independent prognostic factors for OS.

### 3.3 Subgroup Analysis of the EGFR Amplification Group

**FIGURE 4:**
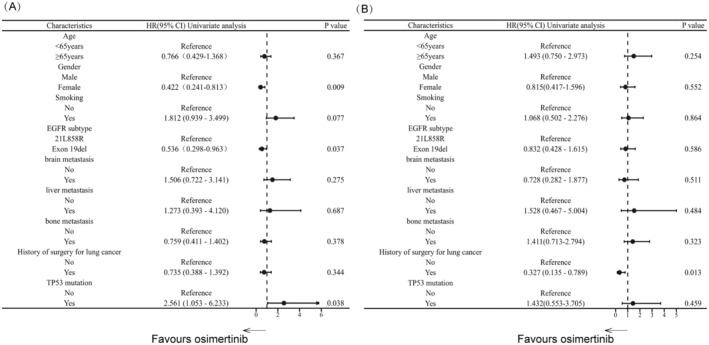
Subgroup analysis of PFS(A) and OS(B) in the EGFR amplification group receiving third‐generation EGFR‐TKI. PFS, progression‐free survival; OS, overall survival.

Among the 59 patients with acquired EGFR amplification and T790M mutation, NGS revealed 53 cases with EGFR‐sensitive mutations, while six cases were identified by ARMS before first‐generation EGFR‐TKI treatment as first‐line therapy. Re‐biopsy and NGS testing were performed after resistance to first‐generation EGFR‐TKI treatment, revealing the retention of the original EGFR‐sensitive mutations in all cases. Among these, 61% (36/59) had EGFR 19del mutation, while 39% (23/59) had EGFR 21L858R mutation. Following monotherapy with the third‐generation EGFR‐TKI osimertinib, patients with EGFR 19del mutation exhibited a significantly longer median PFS compared to those with EGFR 21L858R mutation (17.73 months [95% CI: 12.66–22.80] vs. 9.17 months [95% CI: 4.89–13.44], *p* = 0.034). Moreover, patients with EGFR 19del mutation had a longer median OS (35.77 months [95% CI: 30.31–41.22]) compared to those with EGFR 21L858R mutation (28.73 months [95% CI: 12.22–45.24]), indicating potential clinical benefit, but the difference was not statistically significant (*p* = 0.586) (see Figure 4).

NGS performed before third‐generation EGFR‐TKI therapy revealed that the most prevalent additional concurrent mutation in the EGFR amplification group was TP53 mutation, which was present in approximately 84.7% (50/59) of cases and was mostly attributed to missense mutation. Among patients in the EGFR amplification group, those with and without TP53 mutation receiving third‐generation EGFR‐TKI had a median PFS of 12.07 months (95% CI: 9.837–14.30) and 31.57 months (95% CI: 4.317–58.82), respectively (*p* = 0.033). Their median OS was 28.73 months (95% CI: 17.98–39.48) and 35.87 months (95% CI: 31.66–40.08), respectively (*p* = 0.458). Nonetheless, the number of cases between the two groups was severely imbalanced. Additionally, other concurrent mutations included 2 cases with ERBB amplification, 2 cases with PIK3CA mutation, and 1 case with MET amplification. Given the limited number of carriers for these concurrent mutations compared to non‐carriers, the sample sizes are highly imbalanced. Therefore, we are currently unable to determine the impact of these concurrent mutations on the efficacy of third‐generation TKI in patients with EGFR amplification.

Among patients who developed acquired EGFR amplification after first‐generation EGFR‐TKI treatment, 6.8% (4/59) developed liver metastasis, 18.6% (11/59) developed brain metastasis, and 35.6% (21/59) developed bone metastasis. Following treatment with third‐generation EGFR‐TKI, the median PFS for patients with liver metastasis and those without liver metastasis was 4.07 months (95% CI: 0.00–13.15) and 12.53 months (95% CI: 6.82–18.24), respectively (*p* = 0.667). Their median OS was 7.10 months (95% CI: 0.00–28.35) and 33.90 months (95% CI: 27.50–40.75), respectively (*p* = 0.586). Although patients with liver metastasis had shorter PFS and OS compared to those without liver metastasis, the differences were not statistically significant. The median PFS and OS for patients with brain metastasis receiving third‐generation EGFR‐TKI were 12.07 months (95% CI: 4.05–20.08) and 32.03 months (95% CI: 21.60–42.47), respectively, demonstrating no statistically significant difference compared to those without brain metastasis in terms of median PFS (*p* = 0.273) and OS (*p* = 0.510). Similarly, the median PFS and OS for patients with bone metastasis were 16.53 months (95% CI: 8.61–24.45) and 23.66 months (95% CI: 11.87–35.45), respectively, with no significant statistical difference compared to those without bone metastasis in terms of median PFS (*p* = 0.641) and OS (*p* = 0.319) (see Figure 4).

### 3.4 Survival Analysis of High‐Level EGFR Amplification

Furthermore, we conducted a survival analysis of patients with high‐level EGFR amplification (EGFR CN ≥ 8). Among the 275 patients with advanced NSCLC harboring the T790M mutation, seven patients exhibited acquired high‐level EGFR amplification, while 268 patients were classified as the non‐high amplification (EGFR CN < 8). Our survival analysis revealed that the median PFS for the high‐level EGFR amplification group was 9.17 months (95% CI: 5.33—Not reached, NR), compared to 12.17 months (95% CI: 10.83—12.53) for the non‐high‐level amplification group. Similarly, the median OS for the high‐level amplification group was 23.67 months (95% CI: 14.4—NR), while the non‐high‐level amplification group had a median OS of 24.83 months (95% CI: 22.3—28.73). Notably, there were no significant statistical differences between the two groups regarding median PFS (*p* = 0.277) and OS (*p* = 0.784).

## 4 Discussion

Previous studies have indicated that EGFR amplification may emerge as a resistance mechanism following EGFR‐TKI treatment [20, 21, 24]. However, whether the concurrent presence of acquired T790M mutation and EGFR amplification after resistance to first‐generation EGFR‐TKI impacts the effectiveness of subsequent third‐generation EGFR‐TKI therapy remains uncertain. The current study revealed that patients who developed acquired T790M mutation alongside EGFR amplification after first‐generation EGFR‐TKI resistance demonstrated significantly improved median PFS following treatment with osimertinib compared to those without EGFR amplification (*p* = 0.011). Despite showing potential OS benefits for patients with EGFR amplification, the difference in OS between the two groups was not statistically significant (*p* = 0.164). This lack of significance may be attributed to the relatively small sample size, variability in subsequent treatment regimens, or difference performance status among patients following resistance to third‐generation EGFR‐TKI. Additionally, throughout osimertinib therapy, patients with acquired T790M mutation accompanied by EGFR amplification, particularly those with 19del mutation, exhibited a longer PFS compared to those with 21L858R mutation (*p* = 0.034).

Previous studies have reported a potential association between the concurrent presence of EGFR mutations and EGFR amplification detected before initial EGFR‐TKI therapy and favorable outcomes to EGFR‐TKI treatment. Shan et al. [14] conducted a retrospective study focusing on Asian patients with EGFR‐sensitive mutations who received first‐generation EGFR‐TKI treatment as first‐line treatment. The results suggested that patients with EGFR amplification had a significantly longer PFS compared to those without (16.3 vs. 9.1 months, *p* = 0.004), suggesting EGFR amplification as an indicator for better response to EGFR‐TKI treatment. Another study involving Spanish patients with LUAD demonstrated comparable results, indicating improved responses in patients with EGFR amplification and sensitizing EGFR mutations treated with first‐generation EGFR‐TKI [15]. These findings highlight the potential synergistic role of EGFR amplification and EGFR mutations in tumor development, increasing the molecular dependence of these tumors on the EGFR pathway and potentially improving sensitivity to EGFR‐TKI treatment [14, 15]. However, the role of EGFR amplification may vary based on whether it was acquired before or after EGFR‐TKI treatment. Some studies suggest that EGFR amplification developed during EGFR‐TKI treatment could mediate acquired resistance to EGFR‐TKI. This acquisition may lead to increased mutant allele transcription and EGFR gene activity under EGFR‐TKI treatment pressure [25, 26], manifesting as pronounced genome instability and genome‐wide hypomethylation, ultimately affecting tumor cell sensitivity to EGFR‐TKI [21]. Moreover, acquired EGFR amplification is often accompanied by additional genetic alterations, potentially influencing tumor microenvironment and drug sensitivity [27, 28]. Nonetheless, research on whether the coexistence of acquired T790M mutation and EGFR amplification after resistance to first‐generation EGFR‐TKI affects subsequent third‐generation EGFR‐TKI treatment is lacking.

In the present study, advanced EGFR‐mutant NSCLC patients with acquired T790M mutation and EGFR amplification following resistance to first‐generation EGFR‐TKI demonstrated improved PFS compared to those without EGFR amplification. These findings may be attributed to a proportion of mutated cells transforming into refractory clones through various mechanisms while acquiring resistance after first‐generation EGFR‐TKI treatment, whereas other tumor cells might remain reliant on the EGFR pathway at the molecular level. Hence, subsequent EGFR‐TKI treatment could still exert an anti‐tumor effect in such cases [29]. Furthermore, the emergence of acquired T790M mutation after resistance to first‐generation EGFR‐TKI was found to mediate acquired resistance by sterically blocking the binding of EGFR‐TKI and increasing the affinity for ATP [30, 31]. Third‐generation EGFR‐TKIs have been developed to target the ATP binding site, thereby overcoming this hindrance [32]. Despite the T790M mutation, the activation of the EGFR pathway remains the primary driver of tumor progression. Furthermore, recent studies have indicated that the co‐occurrence of EGFR amplification and EGFR mutations may confer a greater growth and survival advantage to malignant cells compared to either mechanism alone. Both mechanisms activate the EGFR signaling pathway and synergistically contribute to tumor development [14, 15, 16]. Therefore, tumors harboring acquired T790M mutation, acquired EGFR amplification, and EGFR‐sensitive mutations may synergistically amplify the reliance of tumor cells on the EGFR pathway at a molecular level. As a result, these tumors are likely to benefit from treatment with third‐generation EGFR‐TKIs.

However, our findings contradict some previous research results. A phase I study investigating resistance mechanisms to the third‐generation EGFR‐TKI abivertinib in patients with acquired EGFR T790M NSCLC identified three patients with concurrent acquired T790M mutation and EGFR amplification following prior treatment with first‐ or second‐generation EGFR‐TKI. These patients showed resistance to abivertinib [33]. Similarly, two case reports indicated poor response to subsequent third‐generation EGFR‐TKI treatment in patients with T790M mutation accompanied by acquired EGFR amplification detected after resistance to first‐generation EGFR‐TKI [34, 35]. The conflicting findings may be attributed to several factors. Firstly, these studies had a limited number of cases, totaling only five, which compromises the representativeness and generalizability of their findings. Secondly, all patients in these studies harboring both the T790M mutation and EGFR amplification were also found to carry other known mechanisms potentially contributing to resistance to third‐generation EGFR‐TKI, such as MET amplification, high levels of AXL expression, and PIK3CA mutation [36, 37, 38].

TP53 mutation is the most prevalent concomitant mutation in patients with EGFR mutations, accounting for approximately 55%–65% of EGFR‐mutant patients [39]. Moreover, TP53 mutation is also the most common concomitant mutation in patients with EGFR amplification, which is found in approximately 65%–86% of patients with EGFR amplification [27, 40]. Some studies have suggested that the coexistence of TP53 mutation with T790M mutation may be correlated with shorter PFS and OS with third‐generation EGFR‐TKI treatment [28]. In our study, 84.7% (50/59) of patients harboring T790M mutation accompanied by EGFR amplification were found to have TP53 mutation, and patients with TP53 mutation exhibited significantly shorter PFS compared to those without TP53 mutation (12.07 months vs. 31.57 months, *p* = 0.033). Despite the imbalance in sample size between the two groups, these results suggest that concurrent TP53 mutation, T790M mutation, and EGFR amplification may adversely impact the response to targeted therapies.

Notably, monotherapy with third‐generation EGFR‐TKIs has become the most common first‐line treatment option for patients with EGFR mutations. Nevertheless, first‐generation EGFR‐TKIs retain their relevance in specific therapeutic contexts. Studies have shown that combining first‐generation EGFR‐TKIs with bevacizumab as a first‐line therapy results in a PFS of 15.4–17.9 months and an OS of 33.3–50.7 months [41, 42, 43, 44, 45]. In contrast, third‐generation EGFR‐TKI monotherapy typically yields a PFS of approximately 17.8–20.8 months and an OS of about 38.6 months [46, 47, 48, 49]. This suggests that the efficacy of first‐line treatment using first‐generation EGFR‐TKIs combined with angiogenesis inhibitors may be comparable to that of third‐generation EGFR‐TKI monotherapy and could still be a viable option in clinical practice. Moreover, the combination of first‐generation EGFR‐TKIs and bevacizumab does not prevent the development of T790M‐mediated resistance [43, 50]. This indicates that our findings may be beneficial for the subsequent treatment of this patient group.

Nevertheless, the limitations of the current study should be acknowledged. Firstly, the retrospective nature and small sample size may affect the reliability of our findings. Additionally, due to the limited number of patients with PIK3CA mutations and MET amplification in the EGFR amplification group, and the absence of patients with high AXL expression, we are unable to determine whether these concurrent mutations affect the efficacy of third‐generation EGFR‐TKI in patients with T790M mutations and EGFR amplification. Furthermore, the tissue re‐biopsy and genetic testing conducted after first‐generation EGFR‐TKI treatment utilized a small‐panel NGS, which may overlook the influence of undetected mutations on treatment efficacy. Lastly, in the EGFR amplification group, several patients underwent genetic testing by ARMS before initial EGFR‐TKI treatment, raising the possibility of undetected de novo EGFR amplification persisting after first‐generation EGFR‐TKI therapy, although such occurrences may be relatively rare.

In conclusion, contrary to previous findings suggesting acquired EGFR amplification as a predominant mechanism of resistance to EGFR‐TKI, our results demonstrate that after resistance to first‐generation EGFR‐TKI, patients with concurrent acquired T790M mutation and EGFR amplification may benefit from third‐generation EGFR‐TKI, particularly those harboring EGFR 19del mutation.

## Author Contributions

**Yidan Zhang:** data curation, formal analysis, investigation, software, validation, writing – original draft, writing – review and editing. **Yingqi Xu:** data curation, formal analysis, investigation, software, validation, writing – original draft, writing – review and editing. **Jianlin Xu:** formal analysis, software, validation, visualization. **Hua Zhong:** data curation, formal analysis, resources, validation. **Jinjing Xia:** conceptualization, funding acquisition, methodology, resources, supervision, validation, writing – review and editing. **Runbo Zhong:** conceptualization, methodology, project administration, resources, supervision, writing – review and editing.

## Ethics Statement

This retrospective study was approved by the Ethics Committee of Shanghai Chest Hospital (ID: IS24059) and was performed following the Helsinki Declaration of 1964 (revised in 2013).

## Conflicts of Interest

The authors declare no conflicts of interest.

## Supporting information


**Table S1:** 68 genes detected by NGS.
