# Supplementary material for: Osimertinib for EGFR‐Mutant NSCLC Patients With Acquired T790M and EGFR Amplification After First‐Generation EGFR‐TKI Resistance
Source: Cancer Sci. 2024 Dec 31;116(3):753–63. doi: 10.1111/cas.16437 (PMC11875782; doi:10.1111/cas.16437)
Supplement: Supplementary file 1 — Table S1: 68 genes detected by NGS. [file CAS-116-753-s001.doc]

**Supplementary Table S1:**

68 genes detected by NGS.

| ALK | BRAF | EGFR | ERBB2 | KRAS | MET | RET | ROS1 |
| --- | --- | --- | --- | --- | --- | --- | --- |
| AKT1 | APC | ARAF | ATM | AXL | BCL2L11 | BRCA1 | BRCA2 |
| CCND1 | CD74 | CDK4 | CDK6 | CDKN2A | CTNNB1 | DDR2 | ERBB3 |
| ERBB4 | ESR1 | FGF19 | FGF3 | FGF4 | FGFR1 | FGFR2 | FGFR3 |
| FLT3 | HRAS | IDH1 | IDH2 | IGF1R | JAK1 | JAK2 | KDR |
| KIT | MAP2K1 | MTOR | MYC | NF1 | NOTCH1 | NRAS | NRG1 |
| NTRK1 | NTRK2 | NTRK3 | PDGFRA | PIK3CA | PICH1 | PTEN | RAF1 |
| RB1 | SMAD4 | SMO | STK11 | TOP2A | TP53 | TSC1 | TSC2 |
| AR | CYP2D6 | DPYD | UGT1A1 |  |  |  |  |
